# Cardiovascular, perceptual, and performance responses to single- vs. multi-chambered blood flow restriction cuffs

**DOI:** 10.3389/fspor.2024.1469356

**Published:** 2024-11-14

**Authors:** Nicholas Rolnick, Victor S. de Queiros, Masoud Moghaddam, Evan Peikon, Susannah Taylor, Samantha Watson, Campbell Ruffhead, Sean Zupnik, Tim Werner

**Affiliations:** ^1^ Department of Exercise Science and Recreation, CUNY Lehman College, New York, NY, United States; ^2^ The Human Performance Mechanic, New York, NY, United States; ^3^ The BFR PROS, New York, NY, United States; ^4^ Graduate Program in Health Sciences, Federal University of Rio Grande do Norte (UFRN), Natal, Brazil; ^5^ Department of Physical Therapy, University of Eastern Shore, Princess Anne, MD, United States; ^6^ NNOXX Inc., Manson, WA, United States; ^7^ Department of Exercise Science, Salisbury University, Salisbury, MD, United States

**Keywords:** arterial occlusion pressure, B Strong, Delfi, multi-chamber, bladder, resistance training

## Abstract

**Introduction:**

This study aimed to investigate the impact of the blood flow restriction bladder type (single- [SC-BFR] vs. multi-chambered [MC-BFR]) on exercise performance, cardiovascular responses, and perceptual experiences with exercise sessions incorporating multiple sets to volitional failure in a randomized, crossover experimental design.

**Methods:**

Twenty-seven healthy, physically active participants (age: 22.6 ± 5.7; weight: 74.3 ± 15.8 kg; height: 171.7 ± 7.7 cm; BMI: 25.0 ± 4.1 kg/m2; ∼93% reported regular resistance training within 6 months; 11 females) randomly performed exercise to failure (4× sets to failure, 20% 1RM, 1 min rest between sets) in each of three conditions: SC-BFR (using the Delfi Personalized Tourniquet Device inflated to 60% limb occlusion pressure), MC-BFR (using the B Strong Cuffs inflated to 300 mmHg according to manufacturer recommendations), and N-BFR (no BFR control).

**Results:**

SC-BFR blunted post-exercise increases in carotid-femoral pulse wave velocity (*p* = 0.328) (+3.3%) whereas the other conditions showed elevations (MC-BFR +11.8% [*p* = 0.041], N-BFR +9.3% [*p* = 0.012]). Discomfort was lower in N-BFR compared to SC-BFR (*p* < 0.001) and MC-BFR (*p* = 0.035) but all displayed similar exertion (*p* = 0.176). Median total repetitions achieved were significantly less in SC-BFR (57 [25–75th percentile: 47–65) than MC-BFR (76 [63–91] (*p* = 0.043) and N-BFR [106 (97–148)] *p* = 0.005). Per set repetition volumes were similar on set 1 between SC-BFR (*p* < 0.001) and MC-BFR (*p* = 0.001) and were lower than N-BFR (*p* ≤ 0.001) whereas in sets 2–4, MC-BFR performed similar number of repetitions as N-BFR (*p* = 0.984–1.000).

**Conclusion:**

Bladder design of a BFR cuff has an impact on the acute responses to exercise if applied according to recommended application guidelines, as SC-BFR impacts performance to a greater degree and mitigates post-exercise arterial stiffness responses compared to MC-BFR and N-BFR while both BFR conditions display greater levels of discomfort compared to N-BFR.

**Clinical Trial Registration:**

NCT06276673.

## Introduction

Blood flow restriction (BFR) is commonly applied with pneumatic devices capable of determining a relativized individual pressure [known as the limb occlusion pressure (LOP)] (1). Recent research indicates that pressures ≥50% LOP are needed to accelerate muscular fatigue during resistance exercise (evidenced by reductions in repetitions to volitional failure) compared to no BFR, possibly due to enhanced local metabolic stress (2). Therefore, the amount of applied pressure during BFR exercise likely has practical relevance when applied between 40% and 80% LOP using loads between 20% and 30% of the one-repetition maximum.

The growth of BFR in multiple practice settings (3, 4) has spawned numerous cuffs with different device features and characteristics (5). One of the cuff characteristics that has received some attention in the research is the design of the air bladder system. Traditional medical tourniquets capable of relativizing pressure are commonly single-chambered bladder systems with wider cuff widths (6). The single-chambered design functions to provide a circumferential pressure to the limb, reducing and/or potentially eliminating arterial inflow and venous outflow depending on the amount of applied pressure. These bladder systems are the most common type used during practice and have been studied extensively in the surgical and BFR literature (7).

Conversely, a multi-chambered bladder system is not designed to occlude arterial inflow and therefore, relativizing pressures are extremely challenging except on a small number of individuals (8). Multi-chambered bladder systems are composed of numerous sequential bladders separated by small pockets that when fully inflated around the limb, leaves regions of the extremity not under direct pneumatic compression (5). As such, high pressures can be set by the user during application that reduce venous return without significantly compromising arterial inflow, as the set pressure (e.g., the pressure applied by the user) is likely not reflective of the interface pressure (e.g., the pressure applied from the cuff to the user's limb) (8, 9). In lieu of a personalized pressure, the multi-chambered systems recommend starting pressures of between 250 and 350 mmHg for the upper and lower extremities, respectively (10). Given that personalization of pressures is thought to reduce a significant portion of the heterogeneities between different cuffs and BFR methodologies (1), the appearance of a cuff unable to relativize pressure on most individuals has potential significance, especially as research has begun to use the cuff in study designs with important methodological oversights (e.g., misapplying limb circumference algorithms or recommending it for use based upon study designs that do not accurately reflect implementation/efficacy) (5, 11, 12). Moreover, practitioners report using these cuffs in their practice (13). Research is needed to determine whether recommended starting pressures for practicing BFR using multi-chambered devices are efficacious beyond low-load exercise alone as well as determining the relative fatiguability (evidenced by reductions in repetitions to volitional failure) to a single-chambered bladder design.

To the authors’ knowledge, the body of literature is limited to only one study in the upper body that investigated the impact of bladder design on muscle fatiguability. Dancy et al. (14) investigated two single-chambered cuff systems (Delfi Personalized Tourniquet Device [Delfi Personalized Tourniquet Systems, Vancouver, Canada] and SmartCuffs PRO [SmartTools, Ohio, USA]), and a multi-chambered bladder cuff system [B Strong (B Strong Training Systems™, Park City, UT, USA)] during two sets of elbow flexion exercise to volitional failure in healthy adults (14). The results indicate no difference in performance, perceptual experiences, or acute measures of safety between devices, yet some important limitations exist that warrant caution in extrapolation of the findings to practice. First, each person only performed exercise with two cuff conditions, not three. Second, the exercise protocol only included two sets which is not reflective of common practice (1, 4). Moreover, the Delfi Personalized Tourniquet Device reduced repetitions significantly more and had higher rating of perceived exertion in set two compared to the other cuff conditions. It is possible that the two-set design of the study missed an effect that could be elucidated with a 4-set routine commonly used in practice. In addition, it is unknown whether the null effect between cuffs was partially related to the methodology where each participant only received two of the three cuff conditions. Last, as this study was on upper body exercise, it is unknown whether lower extremity exercise exhibits a similar outcome.

Arterial stiffness is a pathological adaptation caused by mechanical stressors resulting in the aggregation of collagen fibers and the gradual loss of elastic proteins in the tunica media layer (15, 16). It is routinely measured in the aorta, brachial, and femoral arteries and typically assessed using the gold-standard method, pulse wave velocity (PWV) (17). PWV measures the speed at which blood pressure waves travel through the arteries, with higher values indicating stiffer, less elastic arteries. Chronic elevations in PWV are an independent risk factor for cardiac events (17, 18). However, limited research exists on the acute PWV response during resistance exercise with BFR and whether this response varies based on the design of the BFR bladder.

Therefore, the purpose of this study was to compare a single- (Delfi Personalized Tourniquet Device) (SC-BFR) vs. multi-chambered (B Strong) (MC-BFR) cuff design on acute performance, perceptual, and cardiovascular (e.g., peripheral and central measures of arterial stiffness) outcome measures during 4 sets of lower body multi-joint exercise to volitional failure using recommended application BFR guidelines. We hypothesized that the SC-BFR would display the largest reductions in repetitions to fatigue followed by the MC-BFR, which would display a greater reduction in repetitions to fatigue compared to free-flow exercise (N-BFR) performed at the same intensity. In addition, we hypothesized that perceptual experiences would be heightened in the SC-BFR and attenuated in the MC-BFR condition but both elevated above N-BFR. Last, we hypothesized that the SC-BFR would blunt the exercise-induced increases in central stiffness compared with low-load exercise as shown in prior research (19). By addressing these questions, this study seeks to provide insights into the practical applications of different cuff designs and their implications for training efficacy and safety.

## Methods

### Participants

Twenty-seven physically healthy and active participants volunteered for the study. Inclusion criteria included 18-40 years of age, weight stable (less than 2.5 kg weight fluctuation in the previous six months), and all female participants were eumenorrheic for at least the last two years. Menstrual patterns were not controlled for due to the nature of the randomization process. Additionally, several recent randomized controlled trials and systematic reviews have called into question the impact of menstrual cycles on vascular compliance (20–23). To qualify for the study, participants also must have met the minimal guidelines for physical activity in the previous six months (24). Exclusion criteria were diagnosis of diabetes, cardiovascular, liver, and/or kidney disease; stage 2 hypertension, sleep apnea, morbid obesity, acute surgery (< 2 months before data collection), and current or past use of tobacco products (25). Each participant signed an informed consent document in accordance with the Declaration of Helsinki acknowledging potential risks and harms. The study was approved by the ethics committee of Salisbury University (protocol #364) and registered at clinicaltrials.gov (NCT06276673).

### Experimental design

**Figure 1:**
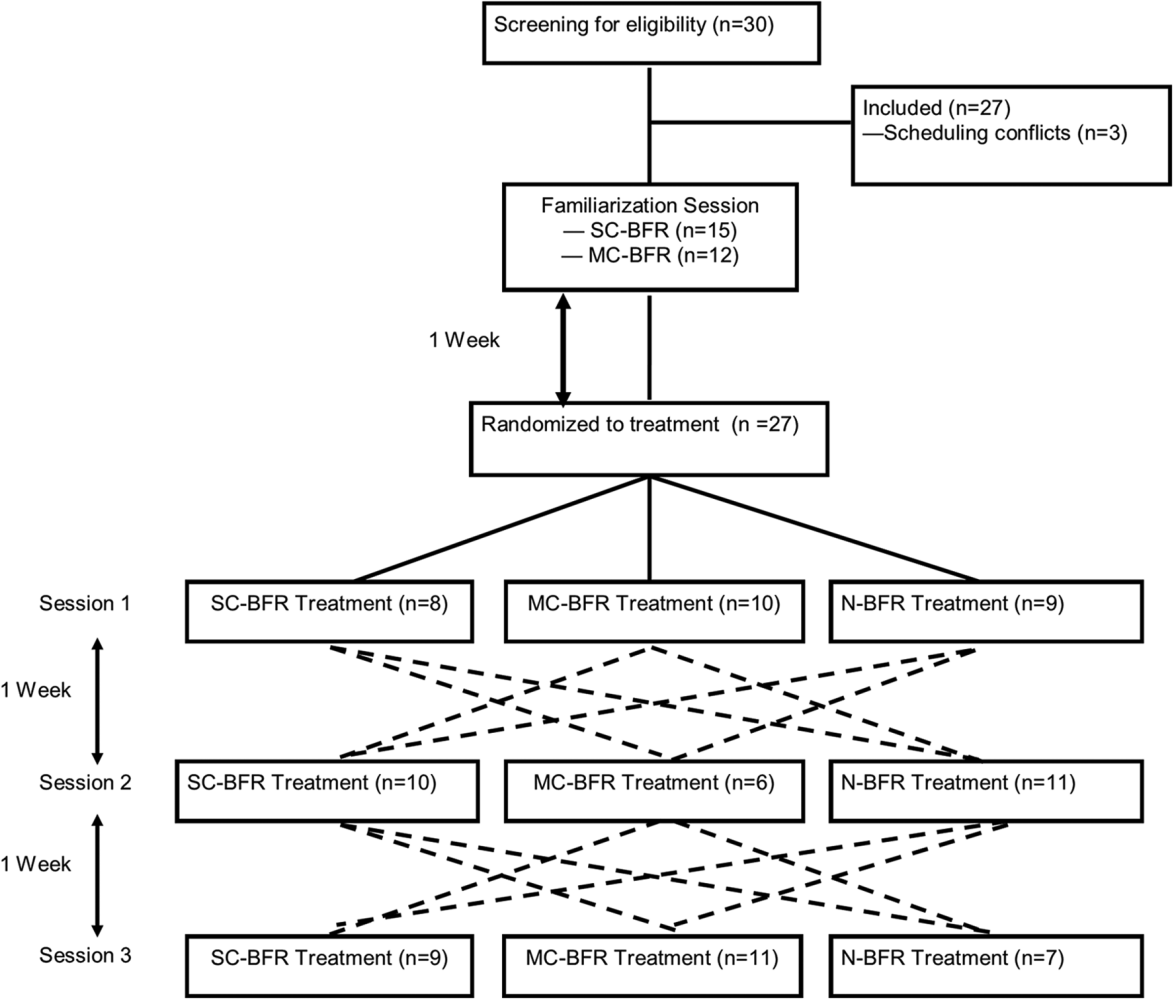
Schematic of all treatment sessions. The dotted lines indicate progression through the trial. For example, where participants undergoing SC-BFR first would then be randomized to either MC-BFR or N-BFR for their second visit and then into the remaining condition for the third visit. The dotted lines represent all possible paths for a participant who underwent the 3-arm trial.

The purpose of the study was to investigate the differences of lower body resistance exercise with or without blood flow restriction (BFR) on acute performance, cardiovascular and perceptual responses. The experimental trials consisted of wall squats with SC-BFR cuffs, MC-BFR, or N-BFR performed to volitional failure. A randomized, crossover design was employed for this study. Randomization was determined by using randomizing software (www.random.org) for all sessions. Participants visited the lab on four separate occasions (Figure 1). The first visit consisted of a familiarization session and an exercise training regime utilizing the BFR cuffs. During subsequent sessions, participants were assigned into one of the three treatments (SC-BFR, MC-BFR, N-BFR) with exercise. Every participant completed all three treatment sessions in a randomized order. Each session, including the familiarization session, was separated by a 1-week washout period and occurred at the same time of day to ensure minimal impact from the previous exposure, to recover from training, and limit diurnal influences. Additionally, all participants were instructed to maintain similar physical activity levels throughout the experiment to reduce the impact of changes in physical activity on the study.

### Procedures

Exercise and data collection occurred in the Exercise Physiology Research Laboratory between 0,700 to 1,300 hrs on the same day each week. To minimize diurnal influences, sessions were also scheduled at the same time of day as the familiarization session. Participants were instructed to continue with their normal exercise routine and dietary regime for the duration of the study, and avoid caffeine, alcohol, and exercise 24 h before each study session. Participants reported fasting for at least 4 h prior to the start of each session.

**Figure 2:**
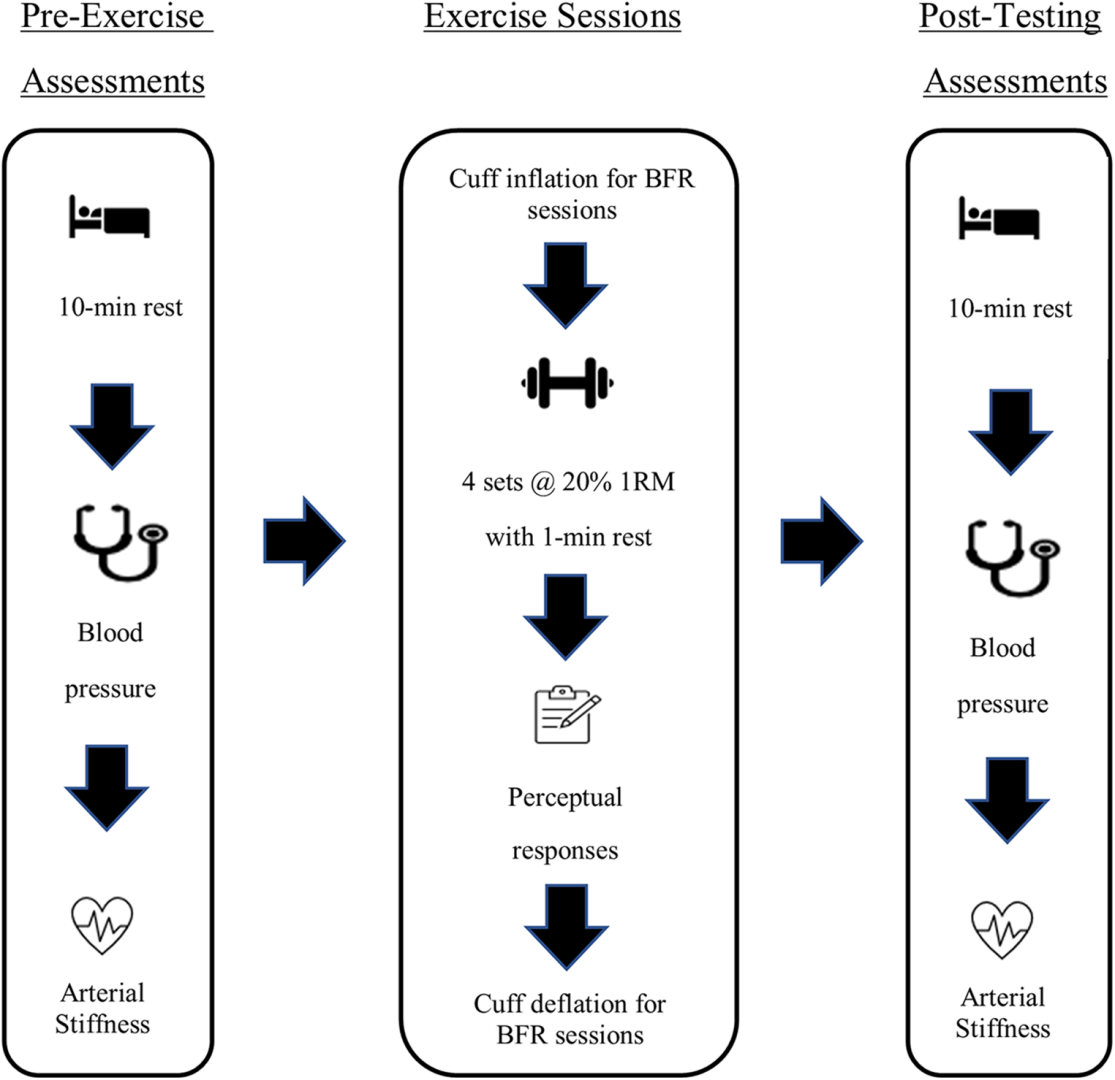
Schematic of the intervention.

**Figure 3:**
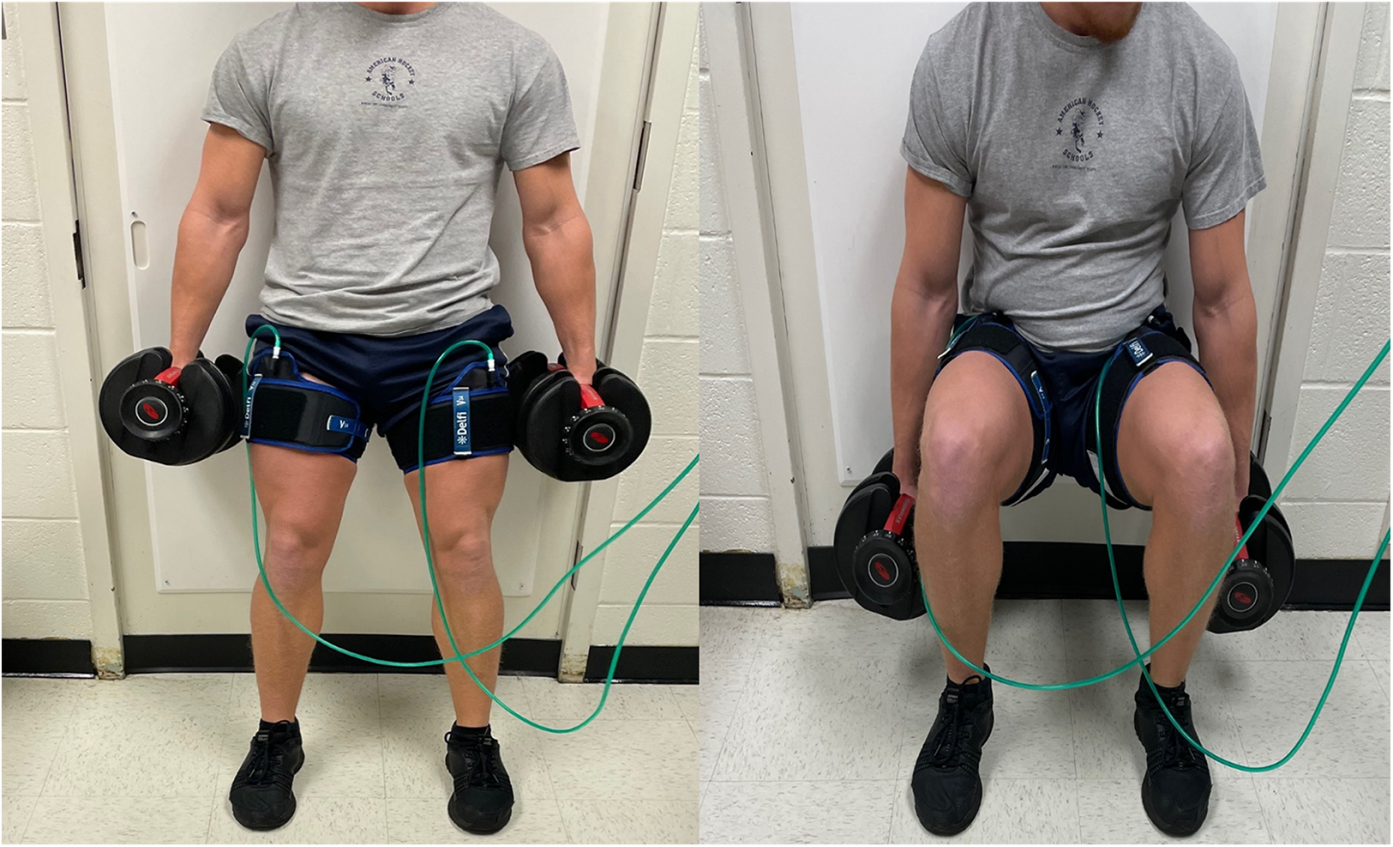
Wall squat performance with the synthetic ice pad mounted on the wall.

All exercise sessions consisted of 4 sets of dumbbell wall squats utilizing 20% of the 1-RM (rounded to the nearest 5lbs increment) to volitional fatigue with a 1-minute rest period between sets (Figure 2). The chosen protocol and load was based upon recommendations from a recent clinical practice guideline (1). Dumbbells were held with shoulder flexed at 0˚ and elbows fully extended throughout the entire exercise. A synthetic ice pad (Snipers Edge, Minneapolis, MN) was mounted on the wall to minimize drag during the downward and upward movement of each repetition (Figure 3). The wall squat range of motion began with knees fully extended followed by a downward movement until a knee flexion of 90˚ was achieved before returning to full extension with the upward movement. Cadence was set at 2 s for both the concentric and eccentric phases, and monitored by a metronome (Seiko, Mahwah, NJ). Participants were instructed to perform the set until volitional failure was reached. Criteria for volitional failure included: participant's desire to terminate the set, inability to maintain appropriate cadence after two violations, and/or the inability to perform the technique according to guidelines (26).

### Anthropometrics

Anthropometric data was collected during the familiarization session. Body height was measured on a stadiometer (Detecto 439 Physician Beam Scale) while the participant stood straight with footwear removed. Total body mass, fat mass, and fat-free mass was measured with air displacement plethysmography (BOD POD) (Cosmed Metabolic Company, Rome). Participants wore tight fitting clothes, were fully voided, and reported fasting for at least 4 h for this assessment. Measurements were recorded in accordance with manufacturer specifications.

### Hemodynamics

After the participant rested in a supine position for 5-10 min, brachial blood pressures (BP) were recorded every two minutes in the right arm using an automated device (Welch Allyn, New York). All BP measurements adhered to American Heart Association guidelines (27). Steady state BP was calculated from the average of three sequential measurements within ±6 mmHg for systolic (SBP) and diastolic (DBP). If after six measurements steady state was not achieved, the last three measurements were averaged and utilized in the calculations.

### β stiffness Index and arterial compliance

After a 10-minute rest in the supine position, longitudinal brightness (B-mode) images proximally 1-2 cm from the right carotid bulb were measured using a multi-frequency linear-array probe (Model 15L4 Smart Mark 4-15 MHz) and doppler ultrasound (Terason t3300, Burlington, MA). Systolic (maximal) and diastolic (minimal) diameters from three cardiac cycles were averaged using device specific software and used in the formulation for β stiffness index (β stiff) and arterial compliance (AC). β-stiff was calculated as β = ln (SBP/DBP)/[(systolic diameter-diastolic diameter)/diastolic diameter] using central BPs (explanation below). Arterial compliance (AC) was calculated as AC = (π(systolic diameter2—diastolic diameter2) ÷ 4(SBP-DBP)). All ultrasound measurements were taken by the same examiner with an intraclass correlation coefficient (ICC) for β stiff of 0.90.

### Pulse wave velocity and central pressures

Acquisition of pulse wave velocity (PWV) was performed by the same examiner with an ICC of 0.93 for PWV. All measurements conformed to manufacture specifications and current guidelines (e.g., approximately 10 min following exercise) (17, 28). After resting in a supine position for at least 10 min, arterial tonometers (Complior Analytic Tonometer, Alam Medical, Vincennes, France) were simultaneously applied on the right carotid, radial, and femoral arteries for the calculation of central [carotid-femoral (cf)] and peripheral [carotid-radial (cr)] pulse wave velocity (PWV). Distance between arterial tonometers were measured using an enlarged caliper to the nearest 5 mm. PWV was calculated as PWV = D (cm)/Δt (sec) after 10 cardiac cycles met the inclusion criteria for the software. Central BP (cSBP and cDBP) was calculated using a device specific algorithm which equated brachial SBP and DBP with carotid pressure waveforms.

### Perceptual responses and performance

RPE, RPD, and a 1–10 Likert scale assessing likelihood of performing the exercise again responses were taken immediately following the last set while the cuffs remained inflated (19). Charts were held up to eye level and questions were asked in the same order: “how hard were you working out?”, “how much discomfort did you feel?”, and “on a scale of 1–10, how likely would you perform the same exercise again? 10 being very likely and 1 being not likely at all.”. Based upon the proximity to the workout, we anticipated collecting peak perceptual experiences given the time it was recorded.

Repetitions (reps) per set and total repetitions for four sets were recorded for each session.

### Familiarization session

Seated blood pressures were the first measurement recorded and conformed to current protocols (27). Height, weight, and body composition followed next. Participants were then provided with instructions on the recording of perceptual responses as discussed in previous interventions (19). After completion of these events, a 1 repetition maximum (1-RM) wall squat test was performed following prescribed guidelines (26). The 1-RM test was only performed once during the entire trial to standardize the loads for the subsequent 3 sessions. A randomized BFR training session with either the SC-BFR or MC-BFR cuffs was then performed utilizing the same exercise protocol as described above. No post-exercise measurements were recorded during the familiarization session.

### BFR device

**Figure 4:**
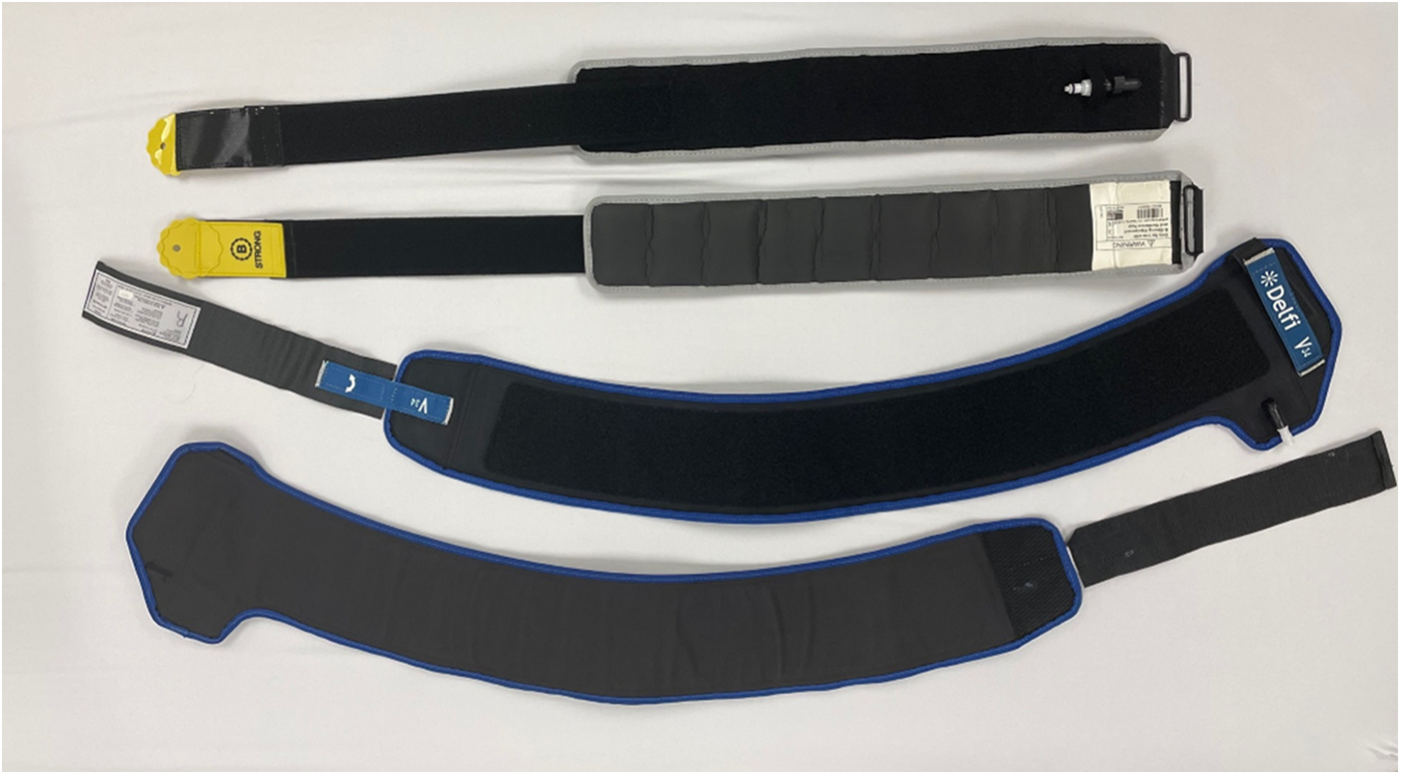
Side-by-side comparison of B Strong (top) and Delfi (bottom) cuffs.

SC-BFR (Delfi, Vancouver, Canada; cuff width, 11.5 cm) and MC-BFR (B Strong, Park City, UT; cuff width, 7.5 cm) training devices were utilized for two of the three exercise sessions (Figure 4). Cuffs were placed around the most proximal potion of the right and left thigh with the participant in a supine position. For the SC-BFR device, 100% of limb occlusion pressure (LOP) was established in supine according to manufacture specifications and pressure was set at 60% LOP for the duration of the session. For the MC-BFR device, pressure was set at 300 mmHg in supine (using the yellow-colored bands designed for lower body use) for the duration of the session based on manufacturer recommendations for initial lower body exercise. Cuff pressure was maintained during the exercise sessions and rest periods for both BFR conditions. Cuff pressure was terminated when perceptual responses were reported after the last set. Because of the nature of the study design, participants were not blinded to treatment. A N-BFR session utilizing the same exercise protocol and intensity without BFR cuffs was also performed in a randomized order.

### Statistical analysis

Data analysis was performed using IBM® SPSS Statistics software (version 24.0). Initially, data normality was explored using the Shapiro-Wilk test. Due to the distribution reported in the perceptual variables, we used Friedman's ANOVA to analyze the effect of the exercise sessions tested on RPE, RPD and likelihood of performing the exercise again. Friedman's ANOVA was also used to analyze the total number of repetitions achieved in the exercise sessions tested. Data from these analyzes are presented descriptively as median and 25th and 75th percentiles. Two-way repeated measures ANOVA was used to analyze the effect of condition and time ([3] conditions × [2] times) for HR, PWV-CF, systolic and diastolic diameter. The sphericity of the data was tested using the Mauchly test; when the sphericity of the data was not met, the Greenhouse-Geisser Correction was performed. The generalized estimating equation (GEE; Gamma distribution model; Autoregressive correlation matrix structure) was used to analyze the effect of time (pre- and post-exercise), condition and interaction for the other variables analyzed. Bonferroni's *post hoc* was used to identify the difference in pairwise comparison. The significance level was set at *p* < 0.05 for all analyses. When the *p*-value was significant, Cohen's d was presented as a measure of effect size for comparisons between means (pairwise comparison). The following classification was used to interpret: Cohen's d: trivial effect (<0.19), small effect (0.20), medium effect (0.50), large effect (>0.80) (29). The r was presented as an effect estimate for data for non-parametric analyses (30). The following classification was used to interpret the magnitude of the r coefficient: small effect (r = 0.10), medium effect (r = 0.30) and large effect (r = 0.50) (30).

## Results

Participant baseline characteristics are shown in Table 1. Thirty participants began the study, but 3 were excluded due to scheduling conflicts. Therefore, twenty-seven participants (11 females) volunteered and completed the study. The cohort's self-identified racial makeup was 66% Caucasian, 26% African-American, and 8% Asian-American. Approximately 93% of the participants (*n* = 25) reported having more than 6 months of resistance training experience (Table 1).

**Table 1:** Participant baseline characteristics.

Variable	Mean ± SD
Age, year	22.6 ± 5.7
Height, cm	171.7 ± 7.7
Weight, kg	74.3 ± 15.8
BMI, kg/m^2^	25.0 ± 4.1
Body Fat,%	16.0 ± 6.3
Fat mass, kg	12.2 ± 5.8
Fat free mass, kg	63.1 ± 13.0
Seated SBP, mmHg	121 ± 11
Seated DBP, mmHg	73 ± 6
Seated MAP, mmHg	89 ± 7
Dumbbell wall squat 1 RM, kg	83.3 ± 42.1
60% LOP RL, mmHg	115 ± 16
60% LOP LL, mmHg	114 ± 15

BMI, body mass index; HR, heart rate; SBP, systolic blood pressure; DBP, diastolic blood pressure; MAP, mean arterial pressure; 1 RM, 1 repetition maximum; LOP, limb occlusion pressure; RL, right leg; LL, left leg.

### Hemodynamic response

There was no effect of condition [W(2) = 1.318; *p* = 0.517], time [W(1) = 0.643; *p* = 0.423] or interaction [W(2) = 2.519; *p* = 0.284] for SBP (Table 2). For pulse pressure (PP), no effect of condition [W(2) = 0.653; *p* = 0.722], time [W(1) = 3.702; *p* = 0.054] or interaction [W(2) = 0.839; *p* = 0.657] was also reported (Table 2). For DBP and MAP, only a time effect was reported (W(1) = 7.676; *p* = 0.006 and W(1) = 5.410; *p* = 0.020 for DBP and MAP, respectively), however, in pairwise comparisons, only a trend was reported between pre- and post-exercise for N-BFR (*p* = 0.059 and *p* = 0.072 for DBP and MAP, respectively) (Table 2).

**Table 2:** Pre- and post-exercise central blood pressure.

	SBP SC-BFR	SBP SC-BFR	SBP MC-BFR	SBP MC-BFR	SBP N-BFR	SBP N-BFR
	Pre-exercise	Post-exercise	Pre-exercise	Post-exercise	Pre-exercise	Post-exercise
Mean	111.93	110.70	111.93	105.15^a^	112.11	105.41^a^
SD	16.93	14.32	13.22	12.97	13.62	13.73
Median	111	107	111	104	116	103
25th	94	99	102	94	103	95
75th	126	124	121	117	125	114
	DBP SC-BFR	DBP SC-BFR	DBP MC-BFR	DBP MC-BFR	DBP N-BFR	DBP N-BFR
	Pre-exercise	Post-exercise	Pre-exercise	Post-exercise	Pre-exercise	Post-exercise
Mean	67.67	66.67	67.11	65.19	66.85	64.56^a^
SD	5.69	6.76	7.27	6.80	6.23	6.13
Median	67	66	67	64	65	65
25th	63	61	63	60	63	59
75th	72	70	72	70	72	69
	MAP SC-BFR	MAP SC-BFR	MAP MC-BFR	MAP MC-BFR	MAP N-BFR	MAP N-BFR
	Pre-exercise	Post-exercise	Pre-exercise	Post-exercise	Pre-exercise	Post-exercise
Mean	82.34	81.26	81.97	78.43^a^	81.86	78.09^a^
SD	8.15	7.61	7.32	7.36	6.58	7.17
Median	81.92	79.59	82.58	78.59	82.25	77.59
25th	75.92	77.26	75.59	72.59	76.26	72.26
75th	91.58	87.25	87.25	82.58	85.58	82.92

SBP, systolic blood pressure; DBP, diastolic blood pressure; MAP, mean arterial pressure; SC-BFR, single-chambered BFR cuff; MC-BFR, multi-chambered BFR cuff; N-BFR, without blood flow restriction; SD, standard deviation.

^a^ Significant difference pre- to post-exercise.

For HR, there was an effect of time [F(1,26) = 126.836; *p* < 0.001] and interaction [F(1.629, 42.348) = 6.485; *p* = 0.006]. No significant differences were reported between HR values reported at baseline (*p* > 0.05), however, post-exercise, N-BFR showed a higher HR than exercise with SC-BFR (Δ = 6.6; *p* = 0.008; d = 0.56). There was no significant difference between the other comparisons performed (MC-BFR vs. SC-BFR and MC-BFR vs. N-BFR). As expected, regardless of the exercise tested, a significant difference was reported between HR reported at rest and post-exercise (*p* < 0.001; Δ = 12.7, Δ = 16.7, Δ = 19.3 for exercise with SC-BFR, MC-BFR and N-BFR, respectively). A time effect was also reported for RPP [W(1) = 126.954; *p* < 0.001]; an increase between pre- and post-exercise values was reported for all exercise conditions tested (*p* < 0.001). However, there was no condition [W(2) = 0.258; *p* = 0.879] or interaction effect [W(2) = 0.928; *p* = 0.629].

There was no effect of condition [W(2) = 0.744; *p* = 0.689] or interaction [W(2) = 4.424; *p* = 0.109] for central SBP, but there was an effect of time [W(1) = 16.131; *p* < 0.001]; there was an reduction in central SBP after MC-BFR (*p* < 0.001; d = 0.52) and N-BFR (*p* = 0.004; d = 0.49), but not after SC-BFR (*p* = 0.602) (Table 2). There was also no effect of condition [W(2) = 1.017; *p* = 0.601] or interaction [W(2) = 0.896; *p* = 0.639] for central DBP, but there was an effect of time [W(1) = 8.796; *p* = 0.003]; a reduction in central DBP was reported after N-BFR (*p* = 0.003; d = 0.37). A time effect was also reported for MAP [W(1) = 19.523; *p* < 0.001]; Central MAP reductions were reported after MC-BFR (*p* < 0.001; d = 0.48) and N-BFR (*p* < 0.001; d = 0.54). On the other hand, there was no effect of interaction [W(2) = 3.437; *p* = 0.551] or condition [W(2) = 1.193; *p* = 0.551] for central MAP. And there were no significant effects of condition or interaction for pulse pressure (all *p* > 0.05) (Table 3).

**Table 3:** Pre- and post-exercise pulse pressure.

	PP SC-BFR	PP SC-BFR	PP MC-BFR	PP MC-BFR	PP N-BFR	PP N-BFR
	Pre-exercise	Post-exercise	Pre-exercise	Post-exercise	Pre-exercise	Post-exercise
Mean	51.52	52.93	51.07	52.63	50.19	50.59
SD	9.47	11.71	9.69	10.19	9.02	8.19
Median	52	51	51	50	50	51
25th	45	47	43	46	45	44
75th	58	62	60	61	58	56

PP, pulse pressure; SC-BFR, single-chambered BFR cuff; MC-BFR, multi-chambered BFR Cuff; N-BFR, without blood flow restriction; SD, standard deviation.

### Pulse wave velocity

**Figure 5:**
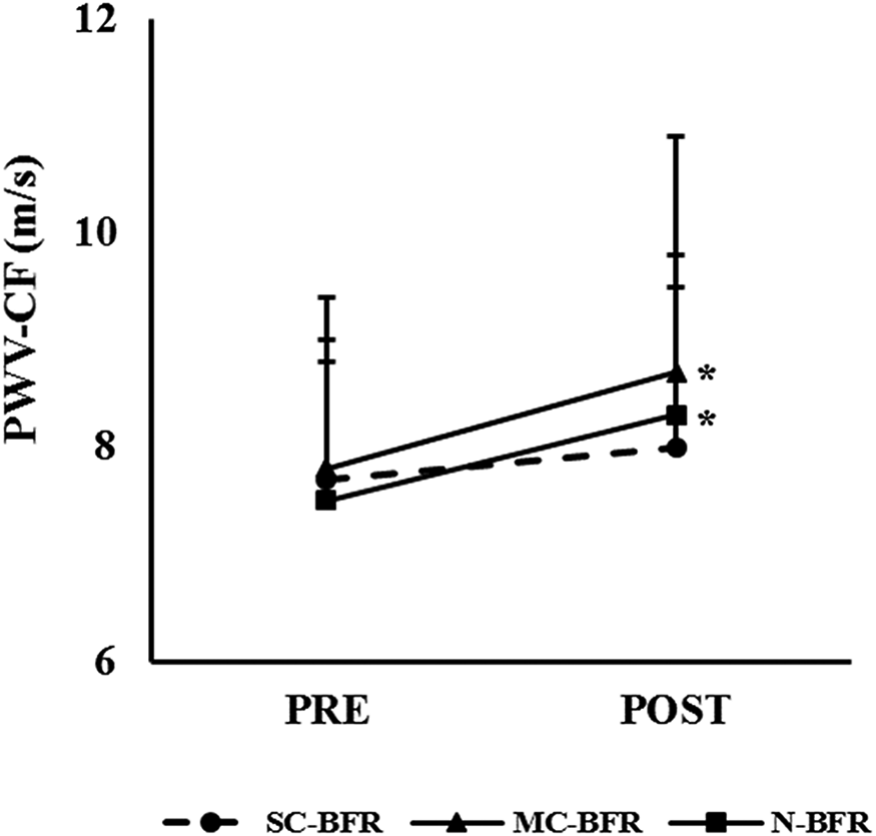
Pre- and post-exercise PWV-CF. * significant difference pre- to post-exercise.

For PWV-CF there was no effect of condition [F (2,52) = 1.1142; *p* = 0.327] or interaction [F (2,52) = 1.360; *p* = 0.266], but there was an effect of time [F (1, 26) = 8.082; *p* = 0.009] (Table 4 and Figure 5). A significant increase was reported in MC-BFR (+11.8%, *p* = 0.041; d = 0.45) and N-BFR (+9.3%, *p* = 0.012; d = 0.56), but there were no significant changes in exercise performed with SC-BFR (+3.3%, *p* = 0.328). For PWV-CR there was no effect of condition [W(2) = 0.853; *p* = 0.650], time [W(1) = 0.099; *p* = 0.752] or interaction [W(2) = 1.956; *p* = 0.376].

**Table 4:** Pre- and post-exercise PWV-CF.

	Mean (SD)	Median (25th—75th)
SC-BFR (Pre-exercise)	10.16 (2.55)	10.1 (8.1–12.4)
SC-BFR (Post-exercise)	10.50 (2.96)	9.9 (8.1–13.3)
MC-BFR (Pre-exercise)	10.51 (1.91)	10.8 (9.1–11.7)
MC-BFR (Post-exercise)	11.27 (3.54)	9.8 (8.9–13.2)
N-BFR (Pre-exercise)	10.08 (2.41)	9.4 (8.1–11.9)
N-BFR (Post-exercise)	11.02 (2.97)	10.4 (8.7–13.2)

PWV-CF, carotid-femoral pulse wave velocity; SC-BFR, single-chambered.

BFR cuff; MC-BFR, multi-chambered BFR Cuff; N-BFR, without blood.

flow restriction; SD, standard deviation.

### β stiffness index and arterial compliance

No effect of condition [W(2) = 1.400; *p* = 0.497], time [W(1) = 0.025; *p* = 0.874] or interaction [W(2) = 5.844; *p* = 0.054] was reported for β Stiffness Index (Table 5). For arterial compliance, no effect of condition [W(2) = 1.070; *p* = 0.586], time [W(1) = 2.050; *p* = 0.152] was reported; an interaction effect was reported [W(2) = 6.824; *p* = 0.033], however, pairwise comparisons adjusted by the Bonferroni correction did not reveal significant differences between the exercise sessions (Table 5).

**Table 5:** Pre- and post-exercise β stiffness index and arterial compliance.

β Stiffness Index						
	SC-BFR	SC-BFR	MC-BFR	MC-BFR	N-BFR	N-BFR
	Pre-exercise	Post-exercise	Pre-exercise	Post-exercise	Pre-exercise	Post-exercise
Mean	4.97	5.71	5.29	4.72	4.61	4.75
SD	1.73	2.91	1.54	1.56	2.07	1.46
Median	4.4972	4.8116	5.0527	4.2640	4.4877	4.7092
25th	3.5553	3.7741	4.3903	3.7271	3.9309	3.3976
75th	5.8724	6.3752	5.7935	5.4818	6.1180	5.8925
Arterial compliance						
	SC-BFR	SC-BFR	MC-BFR	MC-BFR	N-BFR	N-BFR
	Pre-exercise	Post-exercise	Pre-exercise	Post-exercise	Pre-exercise	Post-exercise
Mean	0.00174220	0.00156760	0.00157621	0.00175683	0.00145202	0.00179505
SD	0.000741199	0.000697682	0.000559720	0.000605691	0.000961838	0.000775106
Median	0.00168448	0.00160925	0.00150867	0.00174748	0.00153226	0.00144923
25th	0.00105768	0.00103316	0.00104476	0.00133450	0.00125600	0.00116878
75th	0.00217385	0.00189708	0.00189397	0.00209671	0.00181719	0.00240977

SC-BFR, single-chambered BFR cuff; MC-BFR, multi-chambered BFR cuff; N-BFR, without blood flow restriction; SD, standard deviation.

### Perceptual responses

No significant differences in reported RPE were reported between the exercise sessions tested (*p* = 0.176) (Table 6). In contrast, significance differences were reported for RPD reported in tested exercise sessions (*p* < 0.001). A lower RPD was reported in N-BFR, compared to SC-BFR (*p* < 0.001; r = 0.81) and MC-BFR (*p* = 0.035; r = 0.57). No significant differences were reported between SC-BFR and MC-BFR (*p* = 0.062).

**Table 6:** Perceptual responses reported in tested exercise sessions.

RPE (0–10)			
	SC-BFR	MC-BFR	N-BFR
Median	9	9	9
Minimum	6	5	7
Maximum	10	10	10
Percentiles			
25th	8	8	8
75th	10	9	9
RPD (0–10)			
Median	8	6	^a^5
Minimum	3	1	0
Maximum	10	10	8
Percentiles			
25th	6	5	3
75th	9	8	7
Likelihood to perform again (1–10)			
Median	7	8	9
Minimum	1	1	3
Maximum	10	10	10
Percentiles			
25th	6	6	6
75th	9	10	10

RPE, rating of perceived exertion; RPD, rating of perceived discomfort; SC-BFR, single-chambered BFR Cuff; MC-BFR, Multi-chambered BFR cuff; N-BFR, without blood flow restriction.

^a^ significantly lower than the other conditions.

For likelihood to perform, the Friedman test indicated differences between the exercise sessions tested (*p* = 0.036) (Table 6), however, pairwise comparisons adjusted by the Bonferroni correction did not reveal significant differences between the exercise sessions (*p* > 0.05).

### Performance

Significant differences were reported in the total number of repetitions achieved in the exercise sessions tested in the present study (*p* < 0.001) (Table 7). A lower number of repetitions was achieved in SC-BFR in relation to MC-BFR (*p* = 0.043; r = 0.68) and N-BFR (*p* < 0.001; r = 0.87) (Table 7). Furthermore, a lower number of repetitions was achieved in MC-BFR compared to N-BFR (*p* < 0.001; r = 0.85) (Table 7).

**Table 7:** Total number of repetitions achieved in the tested exercise sessions.

	SC-BFR	MC-BFR	N-BFR
Median	57^a^	76	106
Minimum	28	38	57
Maximum	156	261	307
Percentiles			
25th	47	63	97
75th	65	91	148

SC-BFR, single-chambered BFR cuff; MC-BFR, multi-chambered BFR cuff; N-BFR, without blood flow restriction.

^a^ significantly lower than the other conditions.

**Figure 6:**
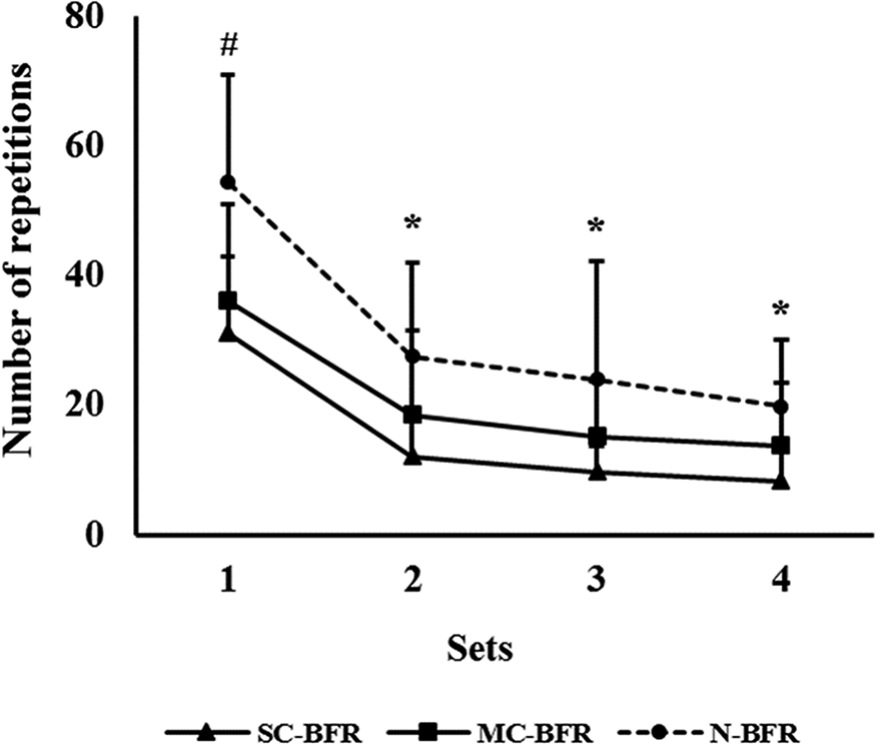
Repetition results from SC-BFR, MC-BFR, and N-BFR trials. ^#^significantly higher number of repetitions in N-BFR compared to MC-BFR and SC-BFR. *significantly higher number of repetitions in N-BFR compared to SC-BFR.

An effect of condition [W(2) = 43.050; *p* < 0.001], time [W(3) = 483.496; *p* < 0.001], but not interaction [W(6) = 12.551; *p* = 0.051] was reported for the number of repetitions achieved in multiple sets (Figure 6). A significantly higher number of repetitions was reported in the first set in N-BFR compared to SC-BFR (Δ = 23.3; *p* < 0.001; d = 1.55) and MC-BFR (Δ = 18.2; *p* = 0.001; d = 1.14). In subsequent sets, no significant differences were reported in the number of repetitions achieved in N-BFR vs. MC-BFR (*p* = 0.984, *p* = 1.000 and *p* = 1.000 for sets 2, 3 and 4, respectively) (Figure 6). In contrast, a higher number of repetitions was reported in N-BFR when compared to SC-BFR in sets 2 (Δ = 15.3; *p* < 0.001; d = 0.97), 3 (Δ = 14.2; *p* = 0.004; d = 0.76) and 4 (Δ = 11.6; *p* < 0.0001; d = 1.06). No differences were reported between SC-BFR and MC-BFR (*p* > 0.05).

## Discussion

This is the second study to compare the performance, perceptual responses, and cardiovascular outcomes between single-chambered and multi-chambered bladder BFR cuff systems and the first in the lower body. The results of our study indicate that (1) largely similar central and brachial cardiovascular and hemodynamic responses between conditions in all assessments except for PWV-CF, central systolic BP, central diastolic diameter, and post-exercise HR which was significantly different to at least one other condition in SC-BFR compared to the other conditions, (2) SC-BFR (Delfi Personalized Tourniquet Device) impacted exercise performance to a greater magnitude than MC-BFR (B Strong Training Systems) with both reducing performance relative to N-BFR; (3) Both BFR cuff systems augmented the discomfort associated with exercise to a similar degree without impacting the exertional effort compared to N-BFR. Therefore, our hypothesis is partially rejected as perceptual demands were similar between SC-BFR and MC-BFR but both induced elevated discomfort compared to N-BFR. Conversely, while both BFR bladder designs impacted performance greater than N-BFR, performance was impacted to a larger extent with SC-BFR, supporting our hypothesis. In addition, increases in post-exercise PWV-CF were blunted in SC-BFR in comparison to the other conditions, providing support for our hypothesis.

### Cardiovascular

This is the second study in the lower body reporting that the Delfi Personalized Tourniquet device blunted post-exercise increases in PWV-CF compared to N-BFR also performed to volitional failure (19). This study adds to the findings of prior research by expanding it to include MC-BFR, which responds similarly to N-BFR control exercise through increasing PWV-CF. Thus, it appears that the blunting of post-exercise increases in PWV-CF is attributable to the autoregulation feature, and not the single-chambered bladder design, as prior research did not show the same blunting effect with the Delfi Personalized Tourniquet cuff configured to a non-autoregulated setting (19). There are several questions that arise with this blunting effect being repeated in a similar cohort. The three most relevant are whether the acute post-exercise blunting of PWV-CF has any clinical significance and whether this effect would be present in other repetition schemes or populations with comorbidities that may present with increased central stiffness at rest. Given the lack of consensus regarding acute changes in PWV-CF on long-term measures of arterial stiffness and health status (31), more research is needed. The current design of this study cannot attempt to answer these questions. One possible explanation could be attributed to the impact of autoregulation, whereby the vasculature is allowed to deform and reform with the varying phases of muscle contraction (32), leading to potentially less sympathetic response during exercise, and thus, blunting of the PWV-CF response. However, this explanation does not provide sound rationale for why N-BFR increased post-exercise PWV-CF. Another possible explanation could be the buffering effect of the autoregulation function through absorption of pulse wave energy, thus attenuating velocity. Future research is needed to determine the relevancy of these observations and potential clinical significance of autoregulation on mitigating central stiffness following exercise.

The other findings of significance are the increased central systolic blood pressure in MC-BFR and N-BFR compared to SC-BFR and the post-exercise HR response being elevated in N-BFR compared to SC-BFR. This finding of no change in central systolic BP supports the impact of autoregulation on mitigating arterial stiffness as increases in central SBP have been associated with elevated PWV-CF (33). As N-BFR produced a superior post-exercise HR change (Δ 6.6 bpm) than the SC-BFR, but no difference between BFR conditions or between MC-BFR and N-BFR, it can be surmised that the reduction of repetitions in the SC-BFR group reduced total workload, and the total heart rate response (34). All other measures responded similarly regardless of the presence of BFR bladder type or strenuous low load control exercise.

### Performance

Our results support the impact of BFR on accelerating repetitions to failure compared to N-BFR, regardless of the air bladder used in the BFR cuff. Our study expands the existing body of BFR literature by showing that the bladder design used in a BFR cuff has an impact on total exercise performance. Specifically, we observed that total median repetitions were significantly lower in SC-BFR (57 repetitions [47–65, 25th to 75th percentiles] across all sets compared to MC-BFR (76 repetitions [63–91, 25th to 75th percentiles] as well as showing that during set 1, both BFR conditions reduced total repetitions similarly (SC-BFR; Δ = 23.3; *p* < 0.001 and MC-BFR; Δ = 18.2; *p* = 0.001) compared to the N-BFR. However, SC-BFR significantly reduced total repetitions across sets 2 (Δ = 14.2), 3 (Δ = 14.2), and 4 (Δ = 11.6) compared to N-BFR while MC-BFR did not produce significant differences in repetitions per set compared to N-BFR. Thus, while no significant differences were observed between BFR bladder designs across the sets between groups, our study supports that the magnitude of repetition loss across each set with SC-BFR is greater than the repetition loss across each set with MC-BFR when performed according to practice (SC-BFR) or manufacturer (MC-BFR) guidelines. This effect may possibly be exacerbated with higher prescribed pressures (70%–80% LOP) in SC-BFR, and should be an area of significant future research interest given we implemented a middle range of % LOP relative to recommended pressures in the lower body (40%–80% LOP) (1).

As this study is the first study comparing repetition loss over multiple sets of lower body multi-joint exercise to volitional failure with different BFR bladder designs, comparison to the existing body of literature is challenging. A prior study utilizing a similar population, randomized crossover design, load, and exercise showed the single-chambered Delfi BFR device that could be set to autoregulation or non-autoregulation performed similarly in mean total repetitions (∼52–53 repetitions) while both reduced repetitions compared to N-BFR (∼83 repetitions) (19). Our findings support a similar magnitude of repetition reduction in our quartile distributions. To date, only one study could be located that has attempted to compare the relative repetition reduction capacity of a multi-chambered device compared to N-BFR control as well as in comparison with single-chambered BFR cuffs, yet had limitations (14). As mentioned prior, this study was performed in the upper body and explored two sets to failure (vs. 4 commonly employed in research and practice) (4, 35) and did not have each participant exercise with all cuff conditions, reducing generalizability. Nonetheless, this study showed that there was significantly greater loss of total repetitions in set 2 in the Delfi Personalized Tourniquet device compared to the B Strong and SmartCuffs (SmartTools, Ohio) in conjunction with higher RPE (14). Thus, we can infer from the set 2 changes that the Delfi device induces greater demands on the exerciser relative to the multi-chambered cuff despite similar total repetitions across the two sets.

Analyzing the distribution of repetitions and repetition loss between different cuff conditions reveals some potentially relevant observations. First, the middle 50% (25–75th quartiles) of our study participants in the SC-BFR and MC-BFR conditions performed between 47 and 65 and 63–91 repetitions, the former of which in totality is less than the commonly recommended 75 repetition protocol used in practice (1). The current design of the study is unable to add clarity to whether the differences observed between BFR conditions would lead to significant differences in longitudinal outcomes of interest (e.g., hypertrophy and strength) in fixed repetition schemes (e.g., 30-15-15-15 reps) and should be investigated in future research. Second, while not significantly different, the absolute range of total repetitions performed by participants between BFR bladder types is large (Δmax—min reps across 4 sets = 128 reps in SC-BFR and Δ = 223 reps in MC-BFR), but both are less than N-BFR (Δ = 297 reps). Future research should explore the intra- and inter-participant variability between bladder types and N-BFR given the spread of participant capacities observed.

### Perceptual responses and safety

Reducing the perceptual demands of BFR exercise has been deemed a barrier to long term compliance (36). Rolnick et al. (36) discussed many ways to potentially increase adherence through modifying the total exercise volume, reducing the pressure applied during training, and deflating between sets, but did not discuss the potential of how the cuff used may impact the perceptual demands of exercise. The current study adds to the existing body of literature indicating that when exercise is conducted to volitional failure, the bladder type used does not appear to significantly impact exertional stress nor exercise induced discomfort, yet both BFR conditions displayed greater median levels of discomfort than N-BFR. Thus, when exercise is conducted to volitional failure, the BFR bladder design does not appear to make a difference in the perceptual demands of exercise. Future research should explore the perceptual demands of fixed repetition schemes to determine if these observations carry over to less strenuous exercise protocols.

The results of the current study are in partial agreement with a recent meta-analysis (37). Our data supports that when taken to volitional failure, RPE between N-BFR and SC-BFR/MC-BFR are similar, but conflict with the discomfort ratings, as N-BFR produced significantly less median levels of discomfort than both BFR conditions. We expected that all conditions would report high levels of RPE given the vigorous exercise intensity (38). However, our data showing no impact of bladder type on discomfort is interesting given the difference in purported blood flow reduction as a result of the applied pressure differences between cuffs (8), and the observed positive relationship between applied pressure and exercise induced discomfort (39). It appears that regardless of the differential pressures applied because of bladder differences between cuffs, BFR exercise-induced discomfort is similar when taken to volitional failure. The reported values in our discomfort ratings were classified as moderate to very high (between 5 and 9 median 25th–75th percentile in both cuff conditions), compared to between 3 and 7 (25th–75th percentiles) in N-BFR (40). As the reported perceptual demands varied significantly in magnitude between all conditions, future longitudinal research is needed to ascertain whether this level of discomfort experienced impacts adherence (36).

Last, we did not observe any adverse events of any kind during or following our investigation. Given the strenuous nature of the protocol, this is surprising given prior research advising avoidance of strenuous exercise with BFR until a short non-failure fixed repetition scheme familiarization session is performed (36, 41). Future research should explore individual and BFR-related factors associated with the occurrence of adverse events.

This study is the first of its kind investigating the impact of BFR bladder type on performance, perceptual responses, and cardiovascular and hemodynamic changes, but it is not without limitations. First, the applied cuff pressures in the SC-BFR and MC-BFR were not relativized according to a similar % LOP. However, the inability to personalize the applied pressure is a design feature of the MC-BFR cuffs (42). Therefore, we implemented manufacturer's recommended pressure settings on the MC-BFR cuff (300 mmHg) as this is what is commonly used in practice and used a moderate % LOP for the SC-BFR condition that is within practice recommendations (42). Future studies could attempt to relativize the applied pressures between cuffs to better isolate the impact of bladder design on acute BFR exercise responses and include supplementary measurements such as total tissue hemoglobin to further elucidate the impact of pressure-dependent relationships between cuffs. Second, we recruited resistance-trained healthy males and females but were likely not powered to assess between-sex differences. Third, while different phases of menstruation have been shown to not impact arterial stiffness measures (20–23), less is known about the perceptual differences. Therefore, caution should be taken when extrapolating the findings to other populations. Fourth, we did not have a sham condition where a cuff is applied but not inflated. Therefore, blinding between conditions did not occur. Future studies should incorporate a sham BFR cuff condition to determine if relevant outcomes are impacted in a similar capacity. Last, the study was on healthy participants, so extrapolating the findings herein to clinical populations should be done with caution.

## Conclusions

The BFR bladder design may impact the acute response to BFR exercise. While both bladder designs applied either according to manufacturer recommendations (MC-BFR) or a relativized pressure (SC-BFR) reduced repetitions to volitional failure compared to N-BFR, the single-chambered system tended to reduce repetitions to a greater magnitude than the multi-chambered cuff. As such, there is the potential for a differential longitudinal response when exercise is performed in fixed repetition scheme protocols, particularly with respect to muscle hypertrophy, as proximity to failure likely differs between cuffs of different bladder constructions performing a similar number of repetitions across multiple sets. In addition, it appears that the single-chambered cuff blunts the central stiffness post-exercise response, although this is likely attributed to the autoregulation function of the Delfi Personalized Tourniquet Device, and not the single-chambered bladder design. Nonetheless, practitioners should consider a single-chambered device if mitigating post-exercise central stiffness is of concern. Last, the addition of BFR—regardless of bladder design—heightens the discomfort associated with exercise to failure over no BFR exercise alone. Practitioners should inform participants to expect higher levels of discomfort compared to N-BFR exercise when performing exercise to volitional failure.

## Data Availability

The raw data supporting the conclusions of this article will be made available by the authors, without undue reservation.
